# Suggestions from Geroscience for the Genetics of Age-Related Diseases

**DOI:** 10.1371/journal.pgen.1006399

**Published:** 2016-11-10

**Authors:** Claudio Franceschi, Paolo Garagnani

**Affiliations:** 1 IRCCS, Institute of Neurological Sciences of Bologna, Bellaria Hospital, Bologna, Italy; 2 DIMES, Department of Experimental, Diagnostic and Specialty Medicine, University of Bologna, Bologna, Italy; 3 C.I.G. Interdepartmental Center "L. Galvani", University of Bologna, Bologna, Italy; 4 CRBA ‐ Applied Biomedical Research Center, S. Orsola‐Malpighi Polyclinic, Bologna; Stanford University School of Medicine, UNITED STATES

## Background

The relationship between aging and major age-related diseases, such as cardiovascular diseases (CVDs), Alzheimer disease (AD), type 2 diabetes (T2D), and cancer and the genetic contribution to both phenomena are important questions in biomedicine. Over the past few decades, each disease has been studied separately in hundreds of genome-wide association studies (GWAS) involving increasing numbers of patients and SNPs, generating results that can explain only in part the genetics of the traits of interest. On several occasions, the results obtained in one population have not been replicated in others, and the clinical application of these results is questionable.

The new field of “geroscience” [1] proposes a conceptual framework that could lead to more effective approaches for studying the genetics of age-related diseases, starting from the basic observation that their main risk factor is age and aging. Geroscience stresses that the basic molecular and cellular mechanisms underpinning aging and its related pathologies are much more interconnected than previously thought on the basis of purely clinical classifications, and largely overlap. This enables us to study and combat the diseases of the elderly all together, rather than one by one [2].

### The Article by Kulminski et al.

**Fig 1:**
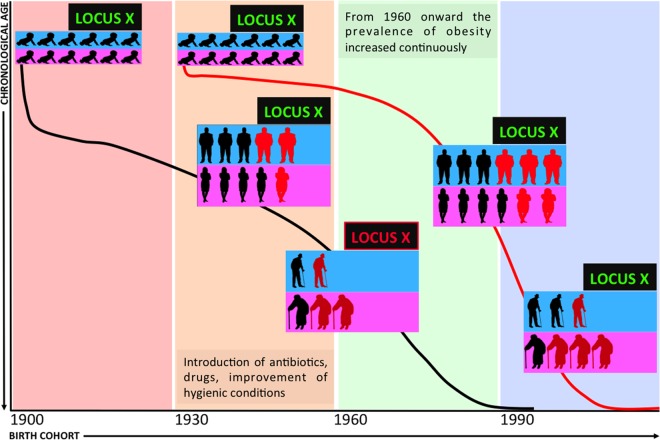
Demography and the genetics of age-related diseases. A schematic representation of the effect of birth cohort and chronological age on the genetic risk of age-related diseases is depicted. The interaction between demographic, environmental, and aging factors allows a genetic risk to emerge (LOCUS green becomes LOCUS X red) only in the presence of a unique combination of chronological age and birth cohort, due to the combined effect of changes in the environmental pressures and the physiopathological remodelling that occurs with age.

The paper by Kulminski et al. [3] provides evidence for how fruitful this approach can be. The authors started by analysing the genetic predisposition to risks of major age-related diseases and mortality, taking advantage of GWAS data generated during the Atherosclerosis Risk in Communities Study (ARCS), and focused on two new promising SNPs (rs222826, rs 222827) on band 2q.22. Using a candidate gene approach, these two SNPs were applied to data from ARCS and from two other studies (Framingham Heart Study and Health and Retirement Study). The combination of advanced statistics and the uniqueness of the datasets, including longitudinal follow-up, allowed them: i) to address the inherent complexity of the genetics of age-related diseases, largely not shaped by natural selection and hidden by age-related heterogeneity and pleiotropic effects of genetic variants [4]; and ii) to explore the causal inferences from selected endophenotypes (body mass index, total and high density lipoproteins). Accordingly, the authors were able to validate risk loci buried in well studied datasets. Notably, in accordance with geroscience, the loci resulted in risks for different age-related diseases, supporting the links between them, and suggesting that this genomic region likely contains elements that play a role in the aging process. In the replication process, the authors noticed that some of the highlighted associations failed to be replicated. Analyzing these apparently contradictory results and profiting from the wealth of available data within these datasets, they found that the association with endophenotypes such as body mass index (BMI) is sensitive to the birth cohort effect, which can be assumed as a rough but informative proxy of environment. This result is in agreement with the intuitive yet neglected idea that genes do not act in isolation, and that observed phenotypes are always the result of gene–environment interactions. This consideration is particularly true for the genetics of age-related diseases, as a given variant interacts with environmental conditions, continuously changing and exerting different selective pressure according to birth cohort (Fig 1). In the last century, pervasive changes in anthropological environments led to significant epidemiological changes. The revolution in hygiene awareness, a major contributor to the unprecedented increase of life expectancy, and the concomitant emergence of an obesogenic environment (easy access to nutrient-rich food; reduced physical activity) exemplify changes that promoted the epidemiological explosion of obesity, metabolic disorders, and eventually of major age-associated diseases. Thus, subsequent generations were exposed to quite different environmental conditions and pressures during the last century, and it is easy to predict that the risk/protective effects of specific alleles changed accordingly. In their causal inference analysis, Kulminski et al. [3] noticed that the correlation of a given allele with a risky endophenotype is also sensitive to chronological age. This result is in line with the antagonistic pleiotropy theory suggesting that a given allele can play different roles at different ages and fits with the remodeling theory of aging [5], according to which the body of each individual undergoes a different or unexpected lifelong process of adaptation to the age-related accumulation of molecular and cellular damages and the consequent functional decline.

Overall, chronological age and birth cohort are central and independent variables that should be carefully considered in studies on the genetics of major age-related diseases. Disregarding such basic demographic variables in cohorts heterogeneous for age and date of birth confounds analysis and results and can contribute to the difficulty in replicating results across different populations [4]. Such difficulties clearly emerge when different cohorts and datasets are put together, including subjects of different ancestry [6], in order to increase statistical power.

### Future Directions

The paper by Kulminski et al. [3] shows how complex the study of the genetics of age-related diseases is in a globalized and changing world. Some people think that a concerted effort to generate whole genome sequences will solve existing problems. This represents a simplistic (and expensive) approach and, on the basis of our experience with GWAS, may have little explanatory or predictive power. The geroscience concept as demonstrated by the work in Kulminski et al., suggests an alternative way forward in which seemingly different phenotypes could have a shared underlying genetic architecture. Important covariates may also be shared, including environment (nutrition, lifestyle, activity, population genetics), sex (since the aging trajectories of men and women are different), and epistatic interactions (including not only the nuclear genome but mitochondrial and microbial genomes) [7–8].

In addition to new computational approaches and efforts, new phenotype models, such as centenarians and their families, could prove extremely useful [9–10] in solving some of the riddles of aging. Time will tell.
